# Desert diversity: genome sequence of *Gordonia rubripertincta* cluster DJ phage Mossy and cluster DV phage Erutan

**DOI:** 10.1128/mra.01245-23

**Published:** 2024-03-12

**Authors:** October Barnes, Christopher J. Workman, Noah C. Patterson, Riley Oesch, Katie L. Johnson, Kaarin Goncz, Joel Sharbrough, Linda C. DeVeaux

**Affiliations:** 1 Department Biology, New Mexico Institute of Mining and Technology, Socorro, New Mexico, USA; Queens College Department of Biology, New York, USA

**Keywords:** actinobacteria, phage, desert

## Abstract

Lytic bacteriophages Mossy and Erutan were directly isolated from desert soil on *Gordonia rubripertincta* and characterized by their morphologies and genomes. Mossy, part of the DJ cluster of Actinobacteriophage, has a genome of 61,183 bp. The genome of Erutan, part of the DV cluster, is 66,957 bp.

## ANNOUNCEMENT

Phages from desert climates are relatively understudied in comparison to marine counterparts (1). We describe the genomes of two novel phages isolated from northern Chihuahuan desert soil.

Mossy and Erutan were isolated from semi-moist soil from New Mexico Tech campus in Socorro, New Mexico (Table 1) on *Gordonia rubripertincta* using standard procedures (2). Soil was suspended in peptone-yeast extract-calcium medium (PYCa), the suspensions filtered (0.2 µm pore size), and plated in PYCa top agar with *G. rubripertincta*. After 3 days of incubation (30°C), a plaque was selected and purified through three rounds of plating. Mossy produced plaques with clear centers surrounded by turbid rings and defined outer borders (diameter ~1 mm; *n* = 10; StDv = 0.05). Erutan produced clear plaques (diameter ~1.5 mm; StDv = 0.04) (https://phagesdb.org/). Both phages exhibited siphovirus morphologies according to transmission electron micrographs (https://phagesdb.org/) analyzed in ImageJ (3). Lysates were placed on 200-mesh formvar-covered, carbon-coated copper grids (EMS) stained with 2% uranyl acetate and imaged in a Hitachi HT7800 TEM at 100 kV. Mossy virions possess a flexible tail measuring 254 nm (*n* = 13; StDv = 38.2) and an icosahedral capsid (width ~66 nm; *n* = 13; StDv = 4.2). Erutan’s capsid was slightly smaller (width ~59 nm; *n* = 4; StDv = 4.1), with a longer tail (length ~404 nm; *n* = 5; StDv = 9.5).

**TABLE 1:** Phage characteristics and genome assembly statistics

Phage	GPS coordinates	Length (bp)	Coverage	GC content (%)	End type	Cluster
Mossy	34.068091 N, 106.909335 W	61,183	1,069×	51.9	Non-redundant terminal repeats	DJ
Erutan	34.067882 N, 106.909707 W	66,957	1,359×	58.4	Circularly permuted	DV

DNA was isolated from high-titer phage lysates using the Wizard DNA Cleanup Kit (Promega, Madison, USA), prepared for sequencing using the NEBNext Ultra II FS Kit, and sequenced on an Illumina MiSeq (v3 Reagents). This yielded ~448K 150-bp single-end reads for Mossy and ~611K 150-bp single-end reads for Erutan. Genomes were assembled using Newbler v2.9, and assemblies were verified using Consedv29 (4) (Table 1). Ends were determined as previously described (4).

Genomes were auto-annotated using Glimmer v3.02b (5) and GeneMarkS 2.5 p (6), and manually verified using PECAAN v20221109 (https://discover.kbrinsgd.org/evidence/summary), BLASTp (7), Phamerator v509 (https://phamerator.org ), and Starterator v509 (http://phages.wustl.edu/starterator). Functions were assigned using BLASTp (against the actinobacteriophages and NCBI non-redundant databases) and HHPred (against the PDB mmCIF70, Pfam-A, and NCBI Conserved Domain databases) (8), and screened for tRNAs by Aragorn (9) and tRNAscanSE (10). Default parameters were used for all bioinformatic analyses (11). Based on gene content similarity of at least 35% to phages in PhagesDB (https://phagesdb.org/), Mossy and Erutan are assigned to phage clusters DJ and DV, respectively (12, 13).

**Fig 1:**
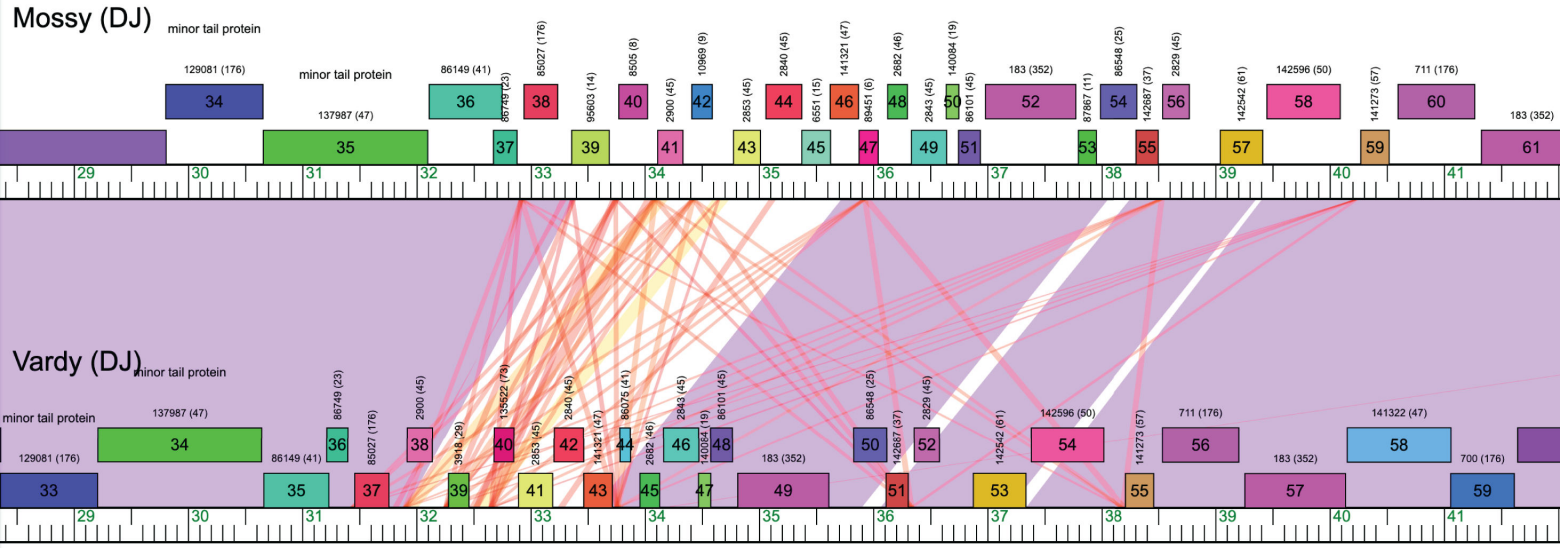
Repeated sequence motif found in cluster DJ phage genomes. Phamerator map of Mossy (top) and Vardy (bottom) phages, with genes indicated by numbered boxes. Purple connecting boxes indicate regions of high BLAST similarity between phage genomes, while white areas indicate sequences not shared across genomes. Red lines indicate short stretches of homologous sequence. In this case, the repeat motif (red lines) is highly abundant in both genomes but variable in its locations. The region with the highest abundance of the repeat motif also coincides with the region of Mossy’s genome that is not shared with Vardy (or other cluster DJ phages).

Mossy’s genome contains 90 predicted genes (25 assigned putative functions). As described for other cluster DJ phages, the middle region contains several repeated instances of a sequence motif that is predicted to be a translational start site (Fig. 1) (14). This repeat appears to be associated with rearrangements based on synteny comparisons with other cluster DJ phages (Fig. 1). Erutan’s genome contains 95 predicted genes (28 with putative functions). One Erutan-specific gene (SEA_ERUTAN_5) aligns best with bacterial sequences (top hit: Caulobacteraceae, NBW15848.1, Max Score = 91.7, *e*-value = 7e−18). Three other cluster members (Gibbin, Tillicus, and Zany) also contain unique, putatively bacterially derived genes at this location, consistent with horizontal gene transfer, perhaps similar to that reported in *Rhodococcus* phages (15).

## Data Availability

Mossy available at GenBank Accession No. OR195046; Sequence Read Archive (SRA) No. SRX20165774. Erutan available at GenBank Accession No. OR475273 and SRA No. SRX20165762. Electron micrographs are available at http://phagesdb.org for Mossy and Erutan.
